# Medical educators’ beliefs about teaching, learning, and knowledge: development of a new framework

**DOI:** 10.1186/s12909-021-02587-x

**Published:** 2021-03-21

**Authors:** Marleen W. Ottenhoff- de Jonge, Iris van der Hoeven, Neil Gesundheit, Roeland M. van der Rijst, Anneke W. M. Kramer

**Affiliations:** 1 Department of Public Health and Primary Care, Leiden University Medical Centre, Hippocratespad 21, Zone V7-P, PO Box 9600, 2300 RC Leiden, The Netherlands; 2 Department of Medicine, Stanford University School of Medicine, Stanford, CA USA; 3 ICLON, Leiden University Graduate School of Teaching, Leiden, The Netherlands

**Keywords:** Teacher beliefs, Beliefs, Conceptions of learning and teaching, Educational beliefs, Educational framework, Faculty development, Framework validation, Orientations to learning and teaching

## Abstract

**Background:**

The educational beliefs of medical educators influence their teaching practices. Insight into these beliefs is important for medical schools to improve the quality of education they provide students and to guide faculty development.

Several studies in the field of higher education have explored the educational beliefs of educators, resulting in classifications that provide a structural basis for diverse beliefs. However, few classification studies have been conducted in the field of medical education. We propose a framework that describes faculty beliefs about teaching, learning, and knowledge which is specifically adapted to the medical education context. The proposed framework describes a matrix in which educational beliefs are organised two dimensionally into belief orientations and belief dimensions. The belief orientations range from teaching-centred to learning-centred; the belief dimensions represent qualitatively distinct aspects of beliefs, such as ‘desired learning outcomes’ and ‘students’ motivation’.

**Methods:**

We conducted in-depth semi-structured interviews with 26 faculty members, all of whom were deeply involved in teaching, from two prominent medical schools. We used the original framework of Samuelowicz and Bain as a starting point for context-specific adaptation. The qualitative analysis consisted of relating relevant interview fragments to the Samuelowicz and Bain framework, while remaining open to potentially new beliefs identified during the interviews. A range of strategies were employed to ensure the quality of the results.

**Results:**

We identified a new belief dimension and adapted or refined other dimensions to apply in the context of medical education. The belief orientations that have counterparts in the original Samuelowicz and Bain framework are described more precisely in the new framework. The new framework sharpens the boundary between teaching-centred and learning-centred belief orientations.

**Conclusions:**

Our findings confirm the relevance of the structure of the original Samuelowicz and Bain beliefs framework. However, multiple adaptations and refinements were necessary to align the framework to the context of medical education. The refined belief dimensions and belief orientations enable a comprehensive description of the educational beliefs of medical educators. With these adaptations, the new framework provides a contemporary instrument to improve medical education and potentially assist in faculty development of medical educators.

**Supplementary Information:**

The online version contains supplementary material available at 10.1186/s12909-021-02587-x.

## Background

The beliefs medical educators hold about teaching, learning, and knowledge determine to a large extent their teaching approaches [1–5]. Because personal educational beliefs drive educators’ behaviour while teaching, these beliefs should be considered a starting point from which to improve the quality of education [6, 7]. Supporting this view, Kember and Kwan stated that fundamental changes to the quality of education rely on changes in educational beliefs [8]. Thus, obtaining more insight into those beliefs is important for the quality of education and may help us to understand why education reform can be cumbersome and faculty development often falls short of changing pedagogical practices [9].

Within the context of higher education a number of studies have explored the educational beliefs of educators and have proposed classification rubrics [2, 6, 8, 10–15]. Such classifications are useful to distinguish between beliefs in a structured way and provide insight into relevant aspects of educational beliefs. However, these classification studies have not been conducted in the field of medical education. Our study addresses a framework that can be used in learning-centred rather than teaching-centred curricula, since currently most medical curricula have adopted learning-centred approaches. We propose a beliefs framework to improve suitability in the context of contemporary medical education.

### Belief orientations

Prior classification studies [2, 6, 8, 10, 11, 13–15] have classified beliefs as global orientations in a continuum, ranging from teaching-centred to learning-centred. While teaching-centred belief orientations focus on the transmission of defined content or knowledge, learning-centred belief orientations focus on students’ conceptual understanding and development. Light and Calkins [15] describe a classification differentiating three belief orientations: teaching-centred, intermediate, and learning-centred. However, they do not base their classification on a fixed set of ‘dimensions’, by which is meant qualitatively different aspects of beliefs. Another classification proposed by Postareff and Lindblom-Ylänne [11] distinguishes 10 different dimensions of beliefs about teaching, learning, and knowledge, structured into four groups. However, this study only differentiates the two belief orientations: teaching-centred and learning-centred.

### Framework of educational beliefs

The primary reason that we chose the framework of Samuelowicz and Bain [14] as the starting point for our study was that in higher education literature their framework is the most extensive, with the broadest scope and content in both belief dimensions and belief orientations. The framework distinguishes seven belief orientations (see Additional file 1). It comprises a two-dimensional matrix ordered according to these belief orientations and belief dimensions. A belief orientation represents a global, composite set of beliefs. In the framework the belief orientations are organised as columns ranging from teaching-centred to learning-centred; qualitatively different belief dimensions appear as rows in the matrix and create distinctions between the belief orientations. Examples of belief dimensions are ‘desired learning outcomes’ or ‘students’ motivation’. Within each dimension a range of beliefs can be distinguished. For example, the beliefs listed within the dimension ‘desired learning outcomes’ are ‘recall of atomised information’, ‘reproductive understanding’, and ‘change in ways of thinking’, respectively, and are ordered on a continuum from teaching-centred to learning-centred. Thus each belief orientation can be further characterised by the belief dimensions. In our opinion, the Samuelowicz and Bain framework’s extensiveness does justice to the complexity and diversity of educators’ beliefs about teaching, learning, and knowledge.

A second reason why we chose this framework as a starting point is how the authors define ‘educational beliefs’. According to their definition, beliefs are ‘typical or characteristic ways’ in which teaching, learning, and knowledge are viewed; they are closely related with practice and contain both cognitive and affective components. These beliefs can only be considered in a holistic way [16]. Thus, the framework describes educators’ deeply rooted, characteristic ways of understanding teaching, and the close relationship between beliefs and practice increases the framework’s usefulness for faculty development interventions.

Finally, the framework uniquely includes a belief dimension related to students’ professional development, which is particularly significant in the context of medical education and has received much attention in recent medical education literature [17–19].

### Research aim and question

The original Samuelowicz and Bain framework was developed in contexts outside of medical education. Therefore, we aimed to adapt this framework to medical education contexts, in order to address the following research question:

What is the content and structure of the beliefs of educators about teaching, learning, and knowledge in medical education?

## Methods

In order to address our research question we conducted a qualitative study using in-depth semi-structured interviews. We interviewed 26 medical educators from two medical schools, all working on preclinical curriculum, with the aim to identify the participants’ characteristic educational beliefs. We used a variety of strategies to enhance the quality of our results [20].

This study is part of a larger research project that explores the longitudinal development of the beliefs of medical educators about teaching, learning, knowledge, and teacher qualities. In this study we report outcomes of the baseline study conducted in 2008–2010 with regard to beliefs about teaching, learning, and knowledge.

### Participants and setting

We opted for a wide variety of participants in our sampling. For this reason, two prominent medical schools from different continents were chosen. We selected 13 faculty members from each school, five teaching basic science topics, five teaching clinical topics and three with roles at the highest educational administrative level with teaching experience, nearly all of them being physicians. One reason for focusing on physicians is that at both SUSM and LUMC, the vast majority of educators are physicians, including those who teach basic science subjects. Another reason is related to the belief dimension about students' professional development. Having a patient-care role next to the other academic roles, we anticipated that physicians may be more likely than non-physicians to model this aspect of the professional identity formation of students. Selection of participants took place on the recommendation of a senior educator and sub-dean from the respective medical schools, and was based on faculty members’ active educational involvement, student evaluations of teaching performance, and teaching awards won by faculty. These selection criteria were chosen because we anticipated that these faculty participants would be most information-rich and their experiences illuminating (*critical case* sampling [21]). With the exception of one educator, all participants had at least 10 years of teaching experience, with an average of 21 years. Only one out of the 26 originally selected medical educators was not able to participate and was replaced by another medical educator who met the criteria.

The two medical schools involved were Stanford University School of Medicine (SUSM), California, USA, and Leiden University Medical Centre (LUMC), Leiden, The Netherlands. Both schools can be classified as research-intensive medical schools and had had their curricula redesigned in the decade prior to the interviews, adopting a more learning-centred approach. Learning-centred curricula have gained popularity worldwide in recent decades and are most common today in medical education.

### Procedure

All interviews were conducted by the first author. We used the interview guide of Samuelowicz and Bain with open-ended questions concerning teaching, learning, and knowledge [14] as a basis for the interview (see interview guide in Additional file 2). Where relevant, we explicitly asked participants to reflect on their small group teaching (classroom instruction within a structured module). Because some literature concludes that educational beliefs can be influenced by class size [22], we wanted to avoid participants answering the questions with exclusively large group lectures in mind. We expected the small group setting to give the most insightful information about participants’ educational beliefs. To ensure that our findings would also be generalizable to other teaching formats, we added questions about whether the participants believed that the teaching format (small group versus large group versus one-on-one teaching) influenced their teaching or the students’ learning. In this way we aimed to gain insight into the beliefs applicable to these educational settings. We also requested that participants focus on their preclinical teaching to rule out differences in course level as an influencing contextual factor, since beliefs may vary according to the level of teaching [13, 22]. Because we aimed to develop a comprehensive holistic image of the beliefs of the faculty participants, we asked them to illustrate their perspectives with examples from their teaching sessions and focused further exploration on the examples that were provided. The interviews of one-hour duration on average were audio-taped and transcribed verbatim. We tested both the interview protocol and the survey questions in a pilot study with three participants who did not contribute to the main study. One of these participants was bilingual. Using their comments, we improved the interview questions and were able to address potential language issues.

### Ethics approval and consent to participate

This study was granted an ethics waiver by the Medical School Ethics Committee of the LUMC (reference C15.033/SH/sh). According to the same committee, formal written informed consent was not required. Similarly, the study was deemed as ‘not involving human subjects research’ by the Stanford University Human Subjects Committee and was exempt from human subjects oversight. The first author invited all the participants by email or telephone, emphasising that participation was voluntary and anonymous. All participants gave oral consent.

### Analysis

For the analysis of the data, we used the original Samuelowicz and Bain framework as a starting point for context-specific adaptation. The analysis consisted of relating relevant interview fragments to the original framework, while we explicitly remained open to ways to modify the framework based on additional beliefs, belief dimensions, or belief orientations identified.

First, each transcript was read and re-read to get an overall sense of the way in which teaching, learning, or knowledge was conceptualised. Then ‘areas of meaning’, text fragments that related to participants’ educational beliefs, were identified. These text fragments were labelled according to the belief dimensions and beliefs of the original framework [14]. Text fragments which did not match an existing belief dimension or belief were given a preliminary code based on the content of the interview fragment. Two team members (IH and MO) analysed each interview independently to enhance *credibility* [20], using Atlas-ti qualitative data analysis software. After the initial coding, the two team members discussed the results. Demarcation rules between the dimensions as well as between the constituent beliefs within each dimension were fine-tuned during the iterative analysis process to enable consistent coding. Parallel to this process we discussed potential new dimensions with their constituent beliefs, grouping and re-grouping the preliminary coded text fragments. Repeated re-coding occurred, and the iterative process continued until all the dimensions and their constituent beliefs stabilised (‘code’ saturation, a technique to improve the *dependability* of the research [23]). This happened after 18 interviews. We frequently negotiated our data together with a third team member (RR) *(investigator triangulation*), looking for evidence and counter-evidence within the data, to reach consensus on all the identified text fragments and to reach agreement as to which dimension they belonged to as well as their constituent belief.

We determined a preliminary belief orientation of the participant holistically, i.e. based on the whole transcript, including all the labelled text fragments. To further confirm data *credibility*, the five interviews which IH and MO considered most difficult to reach consensus on were analysed independently by RR, and the results were negotiated within the research team. The remaining interviews were analysed by both IH and MO, who reached negotiated consensus. During this procedure some minor adaptations were made to belief descriptions. Finally, we re-read all the transcripts again to further ensure data *dependability*, and to confirm ‘meaning’ saturation [23], that is, we checked if we had harvested all the new insights from the data. At the same time, we checked the consistency of the overall belief orientation for each participant, using the final version of the new framework. The process and findings were discussed with the other members of the research team to enhance *confirmability.* Consensus was reached on the adaptation and refinement of the framework.

#### Quality strategies

Two other strategies were used to further confirm the quality of our results. First, we determined an inter-rater agreement of the participants’ belief orientations with the help of an independent research assistant. This assistant was trained in the analysis procedure, using the new framework and codebook, to enable determination of inter-rater agreement *(confirmability by external rater)*. Because the final belief orientations were determined holistically, we decided that we would compare the outcome at the level of the overall belief orientation rather than at the level of text fragments. The inter-rater agreement was determined on 18 interviews through calculation of Cohen’s Kappa.

Second, we provide illustrative interview fragments in the results section, including two narratives of participants with contrasting educational belief orientations (*thick description*, adding to the *transferability* of the findings). We chose one educator holding the most teaching-centred belief orientation (Orientation I), and one holding the most learning-centred belief orientation (Orientation VI).

## Results

### The new framework

Although many beliefs described in the original Samuelowicz and Bain framework were also applicable in a medical education context, our data gave rise to new insights and allowed for refinements. The necessary adaptations in belief dimensions, including their constituent beliefs, and belief orientations will be described below.

The new framework (Table 1) is comprised of six belief orientations set out as columns, and nine belief dimensions set out as rows. The distinct belief orientations have been ordered from left to right according to the degree of learning-centredness and have been numbered from I to VI. The dimensions represent qualitatively distinct aspects of beliefs about teaching, learning, and knowledge. Within each dimension three or four different beliefs can be distinguished. To facilitate the descriptions, beliefs have been categorised as A (teaching-centred), A/b (teaching-centred but with learning-centred aspects), B/a (learning-centred but with teaching-centred aspects), and B (learning-centred). Each of the orientations is thus defined as a unique pattern of beliefs within nine belief dimensions. We have highlighted the divergence from the Samuelowicz and Bain framework in bold (see Table 1).

**Table 1:**
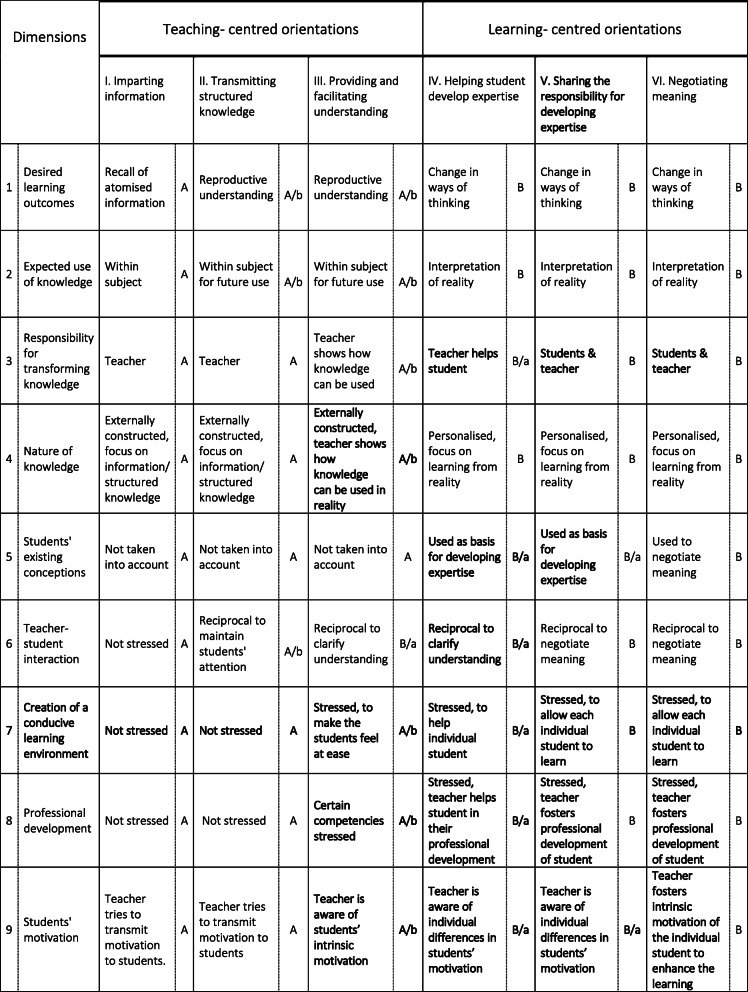
New framework of belief orientations defined by their constituent belief dimensions and beliefs

#### Belief dimensions and their constituent beliefs in the new framework

Table 2 summarises the changes needed within the dimensions. The dimensions adapted to fit our data are presented below in order of numbering.

**Table 2:** Comparison of the dimensions of the new framework and the Samuelowicz and Bain framework

Dimensions:	Comparison of the new framework to the Samuelowicz and Bain framework:
1: Desired learning outcomes	identical
2: Expected use of knowledge	identical
3: Responsibility for transforming knowledge	description of learning-centred beliefs changed
4: Nature of knowledge	extended
5: Students’ existing conceptions	description and categorisation of 2 beliefs changed
6: Teacher-student interaction	categorisation of 1 belief changed
7*: Control of content	removed
7: Creation of a conducive learning environment	new
8: Professional development	extended
9: Students’ motivation	extended

#### Dimension 3: ‘Responsibility for transforming knowledge’

In this dimension we made changes to the description of beliefs to better fit the content of our findings. We identified that in the most learning-centred orientations (Orientations V and VI) the transformation of knowledge was seen as a joint responsibility of teacher and student. In the original framework, this was labelled as the sole responsibility of the student.

#### Dimension 4: ‘Nature of knowledge’

This dimension needed to be expanded from two to three beliefs. We identified a further distinction between the belief of ‘knowledge being externally constructed’. Comparable to the original framework, some educators viewed knowledge as consisting of facts only, described as a ‘database’, as necessary ‘tools’ of factual knowledge, coming from outside sources like books or literature, and not linked to the reality of patient care (coded as D4A).

Others, however, emphasised that this knowledge, even though externally constructed, should be related to patient care by the educator. They believed that the educator should explain how the ‘factual knowledge’ can be used (D4A/b):

*First of all you have basic factual knowledge … I would say the next step is understanding why it is important to possess this knowledge ( … ) that you have to make a kind of doctor’s reflection on it... like when the patient comes with this or that pain or this or that complaint, what is behind it (in basic knowledge);... the focus lies for me on the first steps.* (D4A/b; L12)

#### Dimension 5: ‘Students’ existing conceptions’

This dimension needed an adjustment in the categorisation of the beliefs. In the original framework the transition from a teaching-centred to a learning-centred belief occurs between Orientations IV and V, and in our data between Orientations III and IV. In addition, our participants with a learning-centred belief with teaching-centred aspects (D5B/a) expressed a different aim for involving students’ existing conceptions than described in the original framework. In the original framework, educators with a learning-centred belief with teaching-centred aspects (D5B/a) expressed the aim of preventing common misunderstandings by pointing them out to students and explaining why the established view is more suitable. In our data we found that educators with this belief (D5B/a) indicated instead that they involve students’ existing conceptions to further develop the students’ expertise. Thus, in comparison to the original framework, the emphasis in our findings is less on correcting misconceptions and more on activating preconceptions to develop expertise.

Interviewer: *‘What do the students bring to the learning process?’*

Participant: *‘Knowledge, and experience. ( … ) Indeed, you should … make use of where the student is at this moment and take the next step further to learn new things.’* (D5B/a; L01)

We labelled this fragment a learning-centred belief with teaching-centred aspects (D5B/a), because this participant, unlike participants with a teaching-centred belief (D5A), realises that students are not ‘empty vessels’ but come to a teaching session with their own knowledge and experience. The educator sees it as their responsibility to use and build on these and, together with the students, to further develop knowledge constructs.

#### Dimension 6: ‘Teacher-student interaction’

This dimension contains two learning-centred beliefs (D6B/a and D6B) that differ in the purpose of the teacher-student interaction: clarifying understanding (D6B/a) and negotiating meaning (D6B), respectively. In the original framework, the distinction between these two beliefs resided between Orientation III and IV. From our data we concluded that educators with a Belief Orientation IV, identical to those with a Belief Orientation III, believed that the purpose of the interaction was to clarify understanding. Thus the distinction between the two beliefs (D6B/a and D6B) was placed between Orientations IV and V in the new framework.

#### Dimension 7*: ‘Control of content’

In the new framework this dimension was removed because all of the participants expressed that the control of content was to be determined by the educator. Thus this dimension did not contribute to the classification.

#### Dimension 7: ‘Creation of a conducive learning environment’

We distilled this new dimension from our data, since we discovered that many participants believed that as an educator it is important to create an encouraging learning environment. Educators with the most teaching-centred belief orientations did not stress this or formulated this in a negative way (D7A). Within the most learning-centred orientations, educators explained that they created a positive, personal relationship with individual students to enable them to learn; the focus is on the learning process (D7B):

*I think it’s just really willingness to make the learning fun. You know the students; they like teachers who allow them to learn something new. If teachers just are trying to be nice and popular, they see through that.* (D7B; S02)

In the teaching-centred orientation with learning-centred aspects (Orientation III) the aim of the educator when creating a conducive learning environment is to make students feel at ease (D7A/b), with a focus on the group of students as a whole; in the learning-centred orientation with teaching aspects (Orientation IV), the aim is to help students in their understanding with a focus on the person of the individual student (D7B/a):

*‘To create an atmosphere in which students feel at ease … to freely ask me questions.’* (D7A/b; L12)

*‘I know them as an individual, I care about them and they have a safe place where they can respond.’* (D7B/a; S12)

The main difference between the two learning-centred beliefs (D7B/a and D7B) is the focus of the educator, which is on the person of the student (D7B/a) or on the learning process (D7B), respectively.

#### Dimension 8: ‘Professional development’

In the original framework this dimension consisted of two dichotomous beliefs, while in our data four constituent beliefs could be extracted. Professional development within a medical context can be described as the development of the learner from the role of a student to that of a doctor. In the teaching-centred belief orientations the focus of the teaching is on the academic discipline and less on the professional development of the student. Thus, although some educators recognised the relevance of certain professional competencies, the teaching of these competencies was not primarily aimed at the development of students. The educators with a Belief Orientation I or II believed that students acquired some awareness of these competencies by the educator telling students about them (D8A).

*What I always do is that now and again in the small group is I bring in general knowledge about our healthcare and the market forces … I always try to bring in a few examples. Because I find that students should have a broader helicopter view of healthcare.* (D8A; L05)

The educators with a Belief Orientation III were aware that a variety of professional competencies such as clinical reasoning, collaboration, communication, and professional attitude are important. For these educators, a small group is an appropriate environment to learn these competencies, or they believe some of these competencies can be demonstrated by being a role model (D8A/b).

*… I think that it’s important to work together in small groups; cooperation between students is important … that is important for doctors because they work in teams. So learning to work together, and learning to accept the roles that other people play, because there are often people who take the lead and there are people who hang back. That’s all part of it.* (D8A/b; L12*)*

In the learning-centred belief orientations the focus of the teaching is on the development of the student. Educators within the two most learning-centred belief orientations (Orientations V and VI) described the student’s professional development as an educator’s responsibility and emphasised the importance of fostering the learning or development of the student (D8B). Most participants holding this belief refer to multiple physician roles. In addition to being a clinical expert, the professional physician should also be a communicator, collaborator, leader, health advocate, and scholar (showing qualities such as critical thinking and lifelong learning) [24].

*that if one runs a small group successfully to where people don’t see that they necessarily must be the mirror image of each other but they capitalize on each other’s strengths, then they actually can begin to be learning what they are going to do for a lifetime. So the power of the small group is the power of the professional behaviour that you hope continues forever; especially around team-work and respect – the whole …* (D8B; S05)

Educators within Orientation IV believed that they had an important role in the professional development of students, but were less outspoken about their responsibility in this process (D8B/a).

*For the student, I think that what you especially want is that she/he really participates [in a workgroup]. Maybe it is also something that develops. It would of course be fantastic if after four years, students could take on the role of teacher. One of the things is of course teamwork in the hospital or another place where you work later; so these are skills which are totally essential.* (D8B/a; L01)

#### Dimension 9: ‘Students’ motivation’

This dimension also needed to be expanded from two to four beliefs. In the two most teaching-centred orientations (Orientations l and ll), educators focused on their own interests or enthusiasm, and believed that, consistent with their belief in transmitting knowledge, they also have to transmit their own motivation to the students (D9A):

*I think [teaching] is trying to transmit information in a way that fosters interest in the audience. I think your goal ought to be to generate some excitement, to be excited about what you are teaching, and to be an effective communicator so you can share your excitement, your passion, and generate some enthusiasm in the audience.* (D9A; S07)

In the teaching-centred orientation with learning-centred aspects (Orientation III), educators described their awareness of students’ intrinsic motivation (D9A/b), acknowledging, for example, the enthusiasm of students. In the learning-centred orientation with teaching-centred aspects (Orientation IV) educators were aware of the *individual differences* in students’ intrinsic motivation (D9B/a). These educators used phrases like wanting to learn about what the student is interested in, recognising where the student ‘wants to get’.

*If you consider that intrinsic motivation is most important, then those students who are intrinsically motivated will find their way themselves with a bit of help … I go along with what I think interests them.* (D9A/b; L10)

*try to find something about what’s their driver, what makes them tick, what makes them excited, what makes them feel like it’s worth coming to class. And for some it’s problem-solving, for some it’s knowing something no one else knows … , different reasons, and not assuming that all [reasons] have to be the same.* (D9B/a; S12)

In the most learning-centred orientation (Orientation Vl), educators described their responsibility to find out what makes the learning of the individual student exciting, to invest in the person of the student and his/her passion, and to foster the motivation and interest of the student with the goal of enhancing the learning (D9B):

*What I know about adult learning is that they do best when they are focused on what is important to* *them, and so if they have identified their own specific learning objectives, and we as the facilitator-teacher help them with that, then that is reinforcing and motivating.* (D9B; S11)

#### Belief orientations in the new framework

The original framework’s seventh, most learning-centred orientation, labelled ‘Encouraging knowledge creation’ was not observed in our data. Central to this orientation is the belief that students should be in control of the learning content; none of our participants expressed this belief.

We changed the label of the learning-centred Belief Orientation V from ‘Preventing misunderstandings’ to ‘Sharing the responsibility for developing expertise’. Since our participants with an Orientation V emphasised connecting students’ existing beliefs with the reality of the medical profession rather than correcting misconceptions, this better summarised the pattern of beliefs.

As in the original framework, the medical educators’ focus in Orientations I to III was on the content and its transmission; hence, we conclude that these orientations are teaching-centred. In Orientations IV to VI the focus of the educators was on student learning and development, so we conclude these represent learning-centred belief orientations. The refinements of the dimensions resulted in a sharper demarcation between the orientations. The two belief orientations on either side of the teaching-centred versus learning-centred ‘divide,’ Orientations III and IV, share just one belief. In the original framework these two orientations shared two common beliefs. All other adjacent belief orientations share three to seven beliefs. In the original framework the other adjacent belief orientations shared six to eight beliefs.

### Results of quality strategies

Comparison of the classification of the 18 interviews by the independent research-assistant with that of the authors resulted in a high inter-rater reliability of 0.85 (Cohen’s Kappa). Final consensus was reached for the two interview transcripts that were rated differently. This result validates the framework, supporting that it is not dependent on the perspective of a single educational researcher.

As a final quality strategy we provide a *thick description* of two maximally contrasting belief orientations among our educators (Orientations I and VI). Only one educator in our study displayed an Orientation I; he is a clinician from LUMC. Of the six educators who displayed an Orientation VI we selected the basic science educator to illustrate how a teacher with a learning-centred belief orientation teaches basic science topics. His educational beliefs contrasted with the beliefs of the other basic science educators. This educator worked at SUSM.

#### Dr. A: teaching-centred orientation I: imparting information

In the narrative of Dr. A, the spotlight is on the teacher, who puts a lot of effort into his teaching. What highlights Dr. A’s belief about teaching is his desire to ‘*transfer knowledge*’, which he sees as a tool, and is first introduced from ‘*hardware*’, such as books or electronic information. He emphasises the importance of memorising factual knowledge, as this is a prerequisite for clinical reasoning. *‘You can look everything up, but I don’t think it works like that in practice.’* In his teaching he expects students to be well prepared and checks this by asking questions, for example, about anatomy. Students should be ‘*committed, diligent, and well-behaved’.* He is worried about the attitude and lack of motivation that he observes in some students. He aims to make students take responsibility for working hard by being provocative. For example, he presents a patient case with a bad outcome due to a medical error. In addition to the importance of knowledge transfer, teaching to him means providing students with tips and tricks about how to drill down on the facts. He wants to be a role model, hoping that by demonstrating his own level of knowledge, students will be motivated: *‘( …) what you show then is that you know a lot. It would be very nice if that is motivating for the students. To ensure that you know a great deal about a certain subject.’* A good teacher to him is someone who determines his own teaching goals and achieves them. When asked about what students bring to the learning process, he responds that he is often disappointed that students are so unresponsive. Yet he tries to convey his own motivation on the subject and in this way generate enthusiasm among the students.

#### Dr. B: learning-centred orientation VI: negotiating meaning

In the narrative of Dr. B, students are the main characters, and the focus is on their learning process. He has several aims which he hopes to achieve through his teaching. First that *‘they learn the material’* which is integrated into patient presentations, as *‘they should be able to apply the material to patient care’*. Second, he wants *‘to introduce students to the idea of how they can learn in the future’*. Therefore he spends a significant part of his course analysing medical articles so that students are able to read medical literature and understand its implications for patient care. Third, he aims to teach students to be sceptical and critical and *‘to understand that the literature, the professor, or commonly accepted wisdom can be wrong. So they get a lot of credit for pointing out that I’m wrong.’* His assessments reflect the importance of being able to apply what they have learned: *‘The idea is they have to be able to apply what they learn in the class to the patients and also to be able to extract information from the journal articles that they can apply. [ …]* *If they try to memorise the course notes and take our final exam they won’t do very well, because we ask for synthesis in our final exam.’* Dr. B sees teaching as *‘an alliance, a collaboration between the student and the teacher to learn’.* Knowledge for him is not only about the basic science material, but also about taking care of patients. He emphasises that a lot of reciprocal teaching occurs in his small group setting, and that by splitting the group up into pairs the teaching is *‘completely interactive’.* He is clear that creating a supportive learning environment is a prerequisite for the learning to occur. Thus he emphasises the importance of continuity with the same teacher over a longer period of time in the learning process. *‘They have to learn to trust me, that I won’t make fun of the*m.*’* He places effort into trying to make the learning fun, for example by using competition or games. He sees it as his responsibility to figure out how to engage the students: *‘They come predisposed to learning and the reason is they have a very high incentive to learn because they are really concerned about preparing themselves to take care of patients in the near future and so they have a tremendous incentive to learn. But they’re always demanding to know if it is meaningful or relevant.’*

The narratives as well as the results of the other implemented quality strategies support the utility of the new framework.

## Discussion

Our results confirm the relevance of the structure of the original Samuelowicz and Bain framework in the field of medical education. However, significant changes were required to adapt the framework to a medical education context. We will successively discuss the major changes in the belief dimensions, followed by the consequences for the belief orientations and for the boundaries between the belief orientations.

The most important change in the dimensions is the identification of the new dimension ‘Creation of a conducive learning environment’ (Dimension 7), which was not present in the original framework. We presume that this can be explained by our focusing on small group teaching during the interviews. In this context in which the student is assumed to be actively involved, it is likely that an educator is more aware of the importance of a supportive learning environment. Other medical education literature highlights the importance of ‘nurturing’ or ‘respecting’ students [25, 26], or emphasises the negative impact of learner neglect or frank mistreatment in the learning environment [27, 28]. However, only a single study classifying educational beliefs in the context of higher education [11] has previously recognised the relevance of a conducive learning environment to the educational beliefs of educators.

Secondly, within the dimension ‘Nature of knowledge’ (Dimension 4), we uncovered that a subgroup of medical educators view knowledge as externally constructed, but are also aware that the link to its applicability in the medical profession is important. This awareness may be due to the medical education context, in which most educators are often also involved in clinical work. This most likely encourages educators to link content knowledge to the practice of patient care. Relating meaning to a social reality is an important aspect of the epistemological view of knowledge as being co-constructed [29, 30]. The other relevant aspect of this epistemological view is that a learner conceptualises meaning from interaction with others. This view is also reflected in our data in the belief that the goal of teacher-student interaction is to negotiate meaning (D6B).

Within the dimension ‘Students’ existing conceptions’ (Dimension 5) we did not find support for the belief that students’ misunderstandings should be prevented, in contrast to what was described in the original framework. Instead, we found that some medical educators rather emphasise the importance of building on students’ preconceptions to develop expertise, and sometimes also of learning from students’ conceptions themselves. We hypothesise that this difference might reflect a more current, general awareness that not all preconceptions are misconceptions [31].

Our results lead us to expand the belief dimension concerning the professional development of the student (Dimension 8) from two to four distinct beliefs. Although it is possible that educators in other disciplines show clear beliefs about the development of students, we favour the hypothesis that this emphasis on professional development is due to the context of medical education. Other medical education studies have highlighted beliefs about the professional development of the student. In contrast to our findings, Stenfors-Hayes et al. [32], who compared the beliefs of medical educators working in a preclinical versus clinical context, uncovered the emphasis on the professional development of the students only within the clinical but not preclinical context. In two other studies [25, 33], beliefs about professional development were presented as separate from beliefs about teaching and learning. Our framework, however, integrates these beliefs as one of the dimensions of an educator’s beliefs about teaching and learning.

The addition of two beliefs in the dimension (D9) ‘Students’ motivation’ may have become apparent due to our explicit questions on small group teaching during the interviews. A small group enables an educator to pay attention to the individual student’s intrinsic motivation, which is much more difficult in the setting of large-scale lectures. The most learning-centred belief that an educator should foster the intrinsic motivation of the student (D9B) is in line with other literature which indicates that fostering a student’s intrinsic motivation is associated with deep learning [34, 35].

These refinements add to the descriptive power of the new framework, because the expansion of the constituent beliefs within the dimensions enables a sharper demarcation between the adjacent belief orientations. In the original framework the distinction between some adjacent belief orientations was based on only one belief dimension. Our new framework extends this to at least two dimensions, which makes it possible to determine a medical educator’s belief orientation more reliably.

Significantly, these refinements create a more clearly demarcated boundary between teaching-centred and learning-centred orientations. In the original framework the two adjacent orientations on either side of this boundary (Orientation III and IV, see Table 1) still shared two common beliefs, whereas in our new framework for medical education this has been reduced to only one out of nine beliefs. This is significant as it underlines the sharp boundary between a teaching-centred and learning centred orientation, and reinforces Samuelowicz and Bain’s notion [14] that transition from a teaching-centred to a learning-centred belief orientation means a profound shift in which, according to the new framework, eight out of nine beliefs would be required to change. As the other adjacent belief orientations have three to seven beliefs in common, the differences between these orientations are more subtle, suggesting that these orientations form a continuum. These boundaries may also be easier to cross. In Kember’s review article on teaching beliefs [6], a transitional belief orientation is proposed that would bridge the teaching-centred versus learning-centred orientations. However, our findings, like those of Kember and Kwan [8] as well as Samuelowicz and Bain [14], do not support this bridging belief orientation.

Finally, the disappearance of the dimension ‘Control of content’ led to the absence of the original framework’s most learning-centred belief orientation (Orientation VII). Central to this orientation is the belief that students should be in control of the learning content. We attribute this disappearance to our preclinical context. This conclusion is consistent with the findings of Samuelowicz and Bain [13, 14] who identified this belief exclusively within a postgraduate teaching context.

One other finding merits comment: all beliefs orientations, both learning-centred and teaching-centred, were represented in our sample of participants. That a substantial number of educators did indeed have a teaching-centred belief orientation was surprising, given that the participants selected had been working within a learning-centred curriculum for at least a decade and had extensive experience and deep involvement in teaching. Obviously, in a learning-centred curriculum one would assume a learning-centred orientation to be most effective. Our findings emphasise that the development from a teaching-centred to a learning-centred belief orientation does not automatically take place when a curriculum is innovated towards learning-centredness [36]; more intensive and targeted faculty development interventions may be required.

### Limitations

The data for this study have been collected at two research-intensive medical schools which were both innovating towards a more learning-centred curriculum in the decade prior to the study. The participants’ limited experience with a learning-centred curriculum may limit the generalisability of our results. Research conducted in schools with a longer tradition of learning-centred curricula may reveal further refinements of learning-centred beliefs.

We deliberately selected educators with long-standing teaching experience. This may have created a bias in the distribution of the belief orientations. It is possible that less experienced educators would have displayed more teaching-centred belief orientations. However, all belief orientations were represented in our study.

In addition, the data were gathered a decade ago. It is possible that the beliefs of medical faculty have changed since then, due to new curriculum changes or ongoing faculty development interventions. Yet, the original framework was developed a decade before we conducted our study, and we conclude that in those 10 years the overall structure of the framework remained the same. Moreover, our data show that even a decade after the innovation to a learning-centred curriculum, multiple educators still hold a teaching-centred belief orientation. Thus, we expect that the proposed framework continues to be applicable.

Finally, we intentionally focused on the preclinical teaching context. Therefore, some caution is required when extending conclusions from our findings to other contexts. Indeed, a postgraduate setting, as opposed to our preclinical context, might uncover more learning-centred belief orientations, consistent with the findings of Samuelowicz and Bain [13, 14].

### Implications for teaching and future research

The sharp divide between teaching-centred and learning-centred belief orientations in the framework implies that a change from a teaching- to a learning-centred orientation is a major transition. Current literature emphasises that this transition is a prerequisite for a lasting change in teaching behaviour, which in turn influences student learning. In learning-centred curricula, one way that faculty development programmes could support educators in making this transition is by helping them to become aware of their beliefs. The new framework can make these beliefs more explicit and can encourage medical educators to revisit their beliefs about teaching, learning, and knowledge. The framework’s dimensions can be used as an instrument for reflection and discussion about a medical educator’s educational beliefs. The adaptations and extensions that this framework provides are those areas that are relevant to the context of learning-centred medical education. Reflecting on how to determine which knowledge is relevant to be acquired, how to create a positive learning climate, how to help the students in their professional development, and how to foster intrinsic student motivation, are of major importance for the quality of our education of future health professionals.

Further research is needed to investigate the extent to which the beliefs of medical educators can change and develop towards learning-centredness. For such a study, the presented framework can provide a useful instrument.

## Conclusions

Our study was undertaken to describe and classify the beliefs of medical educators about teaching, learning, and knowledge. Insight into these beliefs is important for medical schools to improve the quality of education and can provide input for faculty development. Although our findings confirm the relevance of the structure of the original Samuelowicz & Bain beliefs framework, developed within higher education research [14], to the field of medical education, we find that significant adaptations and refinements were necessary to align the framework to a medical context. A new dimension ‘Creating a conducive learning environment’ was found, likely uncovered by our explicit questions about small group teaching which aims to encourage a student’s active participation. This emphasis on small group learning likely also explains the new addition of the constituent beliefs in the dimension ‘Students’ motivation’. In addition, the extensions of the beliefs in the dimensions ‘Nature of knowledge’ and ‘Students’ professional development’ are related to the specific medical education context, with its focus on clinical knowledge and professional development. These four new or extended dimensions represent relevant new areas for faculty development. The newly identified and refined beliefs enable a more comprehensive description of the belief orientations of medical educators. As in the original framework, the belief orientations can be arranged on a continuum from teaching-centredness to learning-centredness. Our new framework sharpens the boundary between teaching-centred and learning-centred orientations. With the adaptations proposed herein, the new framework is a more contemporary instrument for faculty development to enable medical faculty to reflect on and revisit their beliefs about teaching, learning, and knowledge.

## Supplementary Information

**Additional file 1.** Samuelowicz & Bain Framework [14].

**Additional file 2.** Interview guide.

## Data Availability

The datasets generated and/or analysed during the current study are not publicly available due to promised anonymity of the participants, but are available from the corresponding author on reasonable request and with permission of the participants in question. The generated codebook is available upon request.

## Abbreviations

LUMC: Leiden University Medical Centre
SUSM: Stanford University School of Medicine
