# Supplementary material for: Medical educators’ beliefs about teaching, learning, and knowledge: development of a new framework
Source: BMC Med Educ. 2021 Mar 21;21:176. doi: 10.1186/s12909-021-02587-x (PMC7981947; doi:10.1186/s12909-021-02587-x)
Supplement: Supplementary file 1 — Additional file 1. Samuelowicz & Bain Framework [14]. [file 12909_2021_2587_MOESM1_ESM.docx]

Additional file 1: Samuelowicz & Bain Framework [14]

| **Dimensions** | | **Teaching- centred orientations** | | | | | | **Learning- centred orientations** | | | | | | | |
| --- | --- | --- | --- | --- | --- | --- | --- | --- | --- | --- | --- | --- | --- | --- | --- |
|  |  | I. Imparting information | | II. Transmitting structured knowledge | | III. Providing and facilitating understanding | | IV. Helping student develop expertise | | V. Preventing misunderstanding | | VI. Negotiating understanding | | VII. Encouraging knowledge creation | |
| 1 | Desired learning outcomes | Recall of atomised information | A | Reproductive understanding | A/b | Reproductive understanding | A/b | Change in ways of thinking | B | Change in ways of thinking | B | Change in ways of thinking | B | Change in ways of thinking | B |
| 2 | Expected use of knowledge | Within subject | A | Within subject for future use | A/b | Within subject for future use | A/b | Interpretation of reality | B | Interpretation of reality | B | Interpretation of reality | B | Interpretation of reality | B |
| 3 | Responsibility for organising or transforming knowledge | Teacher | A | Teacher | A | Teacher shows how knowledge can be used | A/b | Students & Teacher | B/a | Students | B | Students | B | Students | B |
| 4 | Nature of knowledge | Externally constructed | A | Externally constructed | A | Externally constructed | A | Personalised | B | Personalised | B | Personalised | B | Personalised | B |
| 5 | Students' existing conceptions | Not taken into account | A | Not taken into account | A | Not taken into account | A | Not taken into account | A | Used to prevent common mistakes | B/a | Used as basis for conceptual change | B | Used as basis for conceptual change | B |
| 6 | Teacher- student interaction | One-way; Teacher 🡪 students | A | Two-way to maintain students' attention | A/b | Two-way to ensure/ clarify understanding | B/a | Two-way to negotiate meaning | B | Two-way to negotiate meaning | B | Two-way to negotiate meaning | B | Two-way to negotiate meaning | B |
| 7 | Control of content | Teacher | A | Teacher | A | Teacher | A | Teacher | A | Teacher | A | Teacher | A | Students | B |
| 8 | Professional development | Not stressed | A | Not stressed | A | Not stressed | A | Stressed | B | Stressed | B | Stressed | B | Stressed | B |
| 9 | Interest and motivation | Teachers’ | A | Teachers’ | A | Teachers’ | A | Students’ | B | Students’ | B | Students’ | B | Students’ | B |
