# Supplementary material for: Medical educators’ beliefs about teaching, learning, and knowledge: development of a new framework
Source: BMC Med Educ. 2021 Mar 21;21:176. doi: 10.1186/s12909-021-02587-x (PMC7981947; doi:10.1186/s12909-021-02587-x)
Supplement: Supplementary file 2 — Additional file 2. Interview guide. [file 12909_2021_2587_MOESM2_ESM.docx]

Additional file 2: Interview guide

|  | **Main questions** | **Supplementary questions** |
| --- | --- | --- |
| **Questions**  **related to**  **teaching** | Q 1 What do you aim to achieve  through your teaching? | . Do you think you achieve your aims? If not, what happens in practice?  . What, if anything, does prevent you from achieving your aims? |
|  | Q 2 What is teaching? | . Do you think about teaching in this way in all situations? If not, what are other ways in which you think about teaching?  . What is your main concern when teaching? |
|  | Q 3 Does the format of the small group influence your teaching? (as opposed to large group lectures/one-on-one teaching). How? |  |
|  | Q 4 What do you see as your role and as your students' role in the teaching and learning process? | . What are your and students' main responsibilities?  . What do you do apart from telling students about something? |
|  | Q 5 What do students bring to the  learning process? |  |
|  | Q 6 What makes somebody a good  teacher? | . What, if anything, do you see as main obstacles to good teaching? |
| **Questions**  **related to**  **knowledge** | Q 7 What is knowledge in your  discipline? | . Where does knowledge come from?  . Are there different types of knowledge?  . Do you teach different types of knowledge in different ways? How? In particular, how do you teach relationship between theory and practice?  . Do you ever modify your small group-teachings and if so, what does influence the changes you make? |
| **Questions**  **related to**  **learning** | Q 8 What is learning?  Q 9 Does the format of the small group influence the students’ learning? How? | . What does it mean to learn? |
|  | Q 10 How do you know that your students have learned something? | . What are the signs that students have learned something?  . What distinguishes a competent/good student from a poor student?  . If you asked your students at the end of the course 'what have you learned from this course?' what would you like your students to say?  . Do you, and if so how, communicate to students what kind of learning you value? |
|  | Q 11 Do you assess what students have learned through the small group teaching? If so, how? | . What does happen in the teaching process that prepares students for this assessment? |
|  | Q 12 What were the most important ways in which your learning as a student was enhanced? | . How were you made responsible for your own learning? |
| **Question**  **linking teaching and students’ learning** | Q 13 We have talked about teaching and learning; does your teaching influence student learning? How? | . What do you think has the biggest influence on students’ learning?  . What is the most important thing you do in small group teaching that influences ways in which students learn? |
